# The readiness of hospital pharmacists in Kuwait to practise evidence-based medicine: a cross-sectional study

**DOI:** 10.1186/s12911-018-0585-y

**Published:** 2018-01-11

**Authors:** Ali Jasem Buabbas, Fatemah Mohammad Alsaleh, Hamza Mohamad Al-Shawaf, Ali Abdullah, Abdullah Almajran

**Affiliations:** 1 Faculty of Medicine, Kuwait University, PO Box 24923, Safat 13110, Al-Jabriya, Kuwait; 2 Fatemah Mohammad Alsaleh, Pharmacy Practice, Faculty of Pharmacy, Al-Jabriya, Kuwait; 3 Hamza Mohamad Al-Shawaf, MSc Health Informatics and Information Management, Faculty of Allied Health Sciences, Kuwait University, Al-Jabriya, Kuwait

**Keywords:** Pharmacy practice, Evidence-based medicine, Readiness, Hospital pharmacists

## Abstract

**Background:**

The evolving role of pharmacists in providing pharmaceutical care, as part of the healthcare team, challenges them to acquire up-to-date knowledge of medicines to make the best clinical decisions. The volume of medical literature is on the increase, and it is important to utilise these resources to optimise patients’ therapeutic outcomes. This study aimed at assessing the readiness of government hospital pharmacists in practising evidence-based medicine (EBM) in Kuwait in regards to their attitude, knowledge and skills, as well as the perceived barriers and facilitators.

**Methods:**

This descriptive cross-sectional study used pre-tested self-reported questionnaires to collect information from pharmacists working at government hospitals in Kuwait. In addition, one-to-one, face-to-face semi-structured interviews were conducted with the chief pharmacists of all health regions in Kuwait to discuss and identify the barriers and facilitators of implementing EBM in the hospitals. Quantitative and qualitative analytical measures were undertaken for the data acquired from the questionnaires and interviews, respectively.

**Results:**

A total of 176 pharmacists (of 445) working in secondary and tertiary government hospitals in Kuwait agreed to take part in the study, giving a response rate of 40%. Over half of the study sample (*n* = 94, 53.4%) had good confidence in performing online database searches. Approximately 50% of the pharmacists were familiar with searching the Internet for medical resources, asking answerable clinical questions and retrieving research evidence. However, 67% of the pharmacists (*n* = 118) were neither able to apply research evidence to patient care nor capable of identifying knowledge gaps in practice. Barriers to EBM practice were identified, which included limited access to EBM resources (75%), a lack of time and patient overload (71.6%). The interview results confirmed the willingness of the hospital pharmacists to adopt EBM in their practice if necessary resources such as computers and internet connection were provided.

**Conclusion:**

The hospital pharmacists in Kuwait showed good attitude and willingness towards EBM, however, they need to acquire adequate knowledge and skills for applying it in “real life” practise. Using the current results, clinical implications were recommended to demonstrate how to overcome the barriers, wherein hospital pharmacists could be ready to practice EBM.

## Background

The evolving role of pharmacists in providing pharmaceutical care, as part of the healthcare team, challenges them to acquire up-to-date knowledge of medicines to make the best clinical decisions. The volume of medical literature is on the increase, and it is important to utilise these resources to optimise patients’ therapeutic outcomes. Evidence-based medicine (EBM) is defined as “the conscientious, explicit, and judicious use of current best evidence in making decisions about the care of individual patients” [1]. It is considered an approach to integrate recent research evidence into clinicians’ practice, taking into account their clinical experience, patient values and preferences [2]. It aims to improve the decision-making process, which optimises the healthcare outcomes for patients. Therefore, in recent times, healthcare professionals from a variety of medical specialities have increasingly recognised the importance of EBM and have made efforts towards adopting it in clinical practice. This trend is occurring in many countries worldwide, including the United Kingdom, the United States of America, Japan and Indonesia, among others [3–6].

Pharmacy is one of the healthcare professions that plays a pivotal role in the healthcare system. Pharmacists are trained academically to become experts in the proper use and indication of medications. As such, their role can evolve to include assessing, educating and counselling patients to maintain the rational use of medicine and to provide pharmaceutical care to patients [2]. Thus, it is the ethical duty of a pharmacist to provide the best evidence-based information in the provision of pharmaceuticals to patients [7]. The American Association of Colleges of Pharmacy (AACP) [8], the International Pharmaceutical Federation (FIP) and the World Health Organization (WHO) have emphasized on the importance of evidence-based practice for good pharmaceutical care [9]. In this respect, practising EBM requires specific knowledge and skills, which includes determining the clinical question, searching through literature, critically appraising clinical research articles, applying the evidence and lastly, and evaluating the outcomes.

The extant literature includes ample research aimed at investigating physicians’ perception towards using EBM, including barriers to its practice [10–14]. Fewer studies have been published regarding pharmacists and EBM, and most of these primarily relate to community pharmacists, focusing on over-the-counter medications [15–18]. These studies revealed that EBM is a familiar concept among most pharmacists, although they report a number of deficiencies or barriers to its practice [15–18]. For example, a quantitative study conducted in Northern Ireland investigated community pharmacists’ attitudes towards evidence-based practice for over-the-counter medication [15]. The study concluded that pharmacists’ familiarity with evidence-based practice does not necessarily imply that they are ready to practice evidence-based pharmaceutical care. The authors concluded that the mind-set of the pharmacist should be improved in order to acquire the knowledge and skills for good pharmacy practice, and strive to provide evidence-based care [15]. Other studies, including qualitative studies in the United Kingdom have identified several barriers, including pharmacists’ lack of knowledge of EBM or the skills required to utilize the best databases for finding research evidence and critically evaluating it [16–19]. The participants also reported time limitation as a barrier owing to the typically busy schedule of the community pharmacist [17] and lack of evidence of effectiveness of many interventions [16, 17, 19]. Other studies reported barriers specifically associated with community pharmacists, such as being uncomfortable in presenting evidence when the patient asks for treatment advice regarding over-the-counter medications [15, 16]. Therefore, ongoing training for pharmacists in “real life” evidence-based practice was recommended in almost all related studies [15, 18, 19].

### Current EBM research in the region

In the Gulf region, EBM practice in healthcare organisations is at an early stage. Most studies in the region have been conducted to assess the attitude and awareness of physicians towards EBM. It was found that while physicians’ attitude towards EBM is generally positive; important barriers exist, as reported by studies in Iran [12], Dubai [13], Saudi Arabia [14], Bahrain [20], Oman [21] and Qatar [22]. In Kuwait, two similar studies have been published, one among primary care physicians [23] and the other among dentists [24]. Both these studies reported that the overall awareness of physicians and dentists towards EBM practice was low. The studies recommended that both physicians and dentists should be provided with free access to medical databases and training courses.

In contrast, scarce data exists on the use of EBM concepts in hospital pharmacy practice in the Gulf region or the Middle East [2, 7]. A recent study was conducted in Jordan to assess the awareness, knowledge and skills of pharmacists regarding the practice of EBM [7], reporting a positive attitude among hospital pharmacists towards EBM, and identifying individual and organisational barriers towards its practice.

In Kuwait, pharmacy education is offered mainly through a Bachelor of Pharmacy degree. More recently, a Master degree in Pharmaceutical Sciences has been initiated. However, strong training in EBM is not included in either curricula. In clinical practice, hospital pharmacists practise numerous tasks that require them to have up-to-date knowledge of medications in order to answer questions raised by patients, physicians or any other healthcare practitioner. The clinical experience of the pharmacist is not always adequate to provide the best pharmaceutical care. However, by using the most up-to-date evidence from the literature together with experience, the pharmaceutical care can be improved [25].

Based on the literature review, it is important to investigate the factors necessary for practising EBM, as each healthcare profession has its own specific nature and needs. In this respect, the current study focuses on assessing the readiness of hospital pharmacists in Kuwait towards practising EBM.

### Research question

Are pharmacists in government hospitals in Kuwait ready to practise EBM?

### Aim and objectives

This study aimed to assess the readiness of the pharmacists working at government hospitals in Kuwait to practise EBM by: 1) assessing the pharmacists’ current knowledge and skills in relation to EBM; 2) exploring the pharmacists’ opinions and attitude towards practising EBM; and 3) identifying the perceived barriers and facilitators with regards to the practice of EBM in the hospital pharmacies.

## Methods

This descriptive cross-sectional study was conducted among pharmacists working in the government hospitals in Kuwait. Preliminary fieldwork showed that there were a total of 445 pharmacists working in the government hospitals located in all six governorates of Kuwait. One secondary hospital situated in each governorate provides general medical care for that region (Ahmadi, Farwaniya, Jahra, Al-Sabah, Hawalli and Capital), and 22 tertiary hospitals located in the Al-Sabah Health Region provide specialised medical care. Private hospitals in Kuwait were excluded from the current study since they have criteria and quality policies for recruiting pharmacists that differ from government hospitals.

A self-administered 85-item questionnaire was used to conduct the current study. The questionnaire comprised of four sections: 1) demographic characteristics (11 items); 2) knowledge and skills for EBM (22 items); 3) opinions and attitude towards EBM (30 items); and 4) barriers and facilitators for EBM practice (22 items). All questions were close-ended and presented as “select the appropriate answer”. The questions in sections 2 to 4 were provided with either a four-point or a five-point Likert scale.

The questions had been tested for reliability in previous studies [7, 26]. Slight modifications were made either to clarify some of the questions (without changing their essence) or to collect more data. Five pharmacists pretested the questionnaire to ensure the clarity and relevance of the questions. Since the majority of the pharmacists graduated from English-only programs, an English language questionnaire was used. The questionnaires were distributed in-person to all available hospital pharmacists during their duty shifts, and completed questionnaires were collected on the same day, or the day after by the same researcher. Participants were selected by convenience at the time the researchers visited each centre. A second visit to the research setting was considered to collect the completed questionnaires and to invite more participants to enhance the response rate.

Within each of the six health regions, a chief pharmacist functions as the main representative of regions and the higher management in the Kuwait Ministry of Health (MOH). Individual, face-to-face semi-structured interviews were conducted by a trained researcher with all six chief pharmacists to discuss and identify the barriers and facilitators of implementing EBM in the hospitals. Chief pharmacists, in addition to their supervisory duties, are responsible in developing guidelines and regulations in their healthcare regions.

Interview questions were formulated by the research team after reviewing the literature of similar studies [13]. The interview content was topic guided with probes to motivate the interviewee to provide more details about the topic, and was comprised of questions on three main themes: the overall concept of EBM, the adoption of EBM in pharmacy practice and factors that facilitate or hinder the implementation of EBM in pharmacy practice in Kuwait.

The chief pharmacists were approached and interviewed in their offices by one of the authors who had experience in conducting the interviews. Mentioning the reason of the visit made the chief pharmacists willing to participate in this study. The interviews were noted manually due to the preference of most of the interviewees, who did not accept audio recording of the interview. Upon commencing the interview, the definition of EBM was introduced verbally to the interviewees.

Prior to starting the data collection, the aim of the study was explained verbally to the participants, who were also informed that their responses will be anonymous and used only for research purposes. Written Informed Consent was obtained from all participants. Ethical approval was obtained from the Ethical Research Committee at the MOH, Kuwait. The data collection procedures were undertaken for over a period of three months.

### Statistical analysis

Data from the questionnaires were analysed using Statistical Package for the Social Sciences (SPSS) version 24. Frequency tables for all variables and percentages for each group were calculated. The chi-square test was used to test the statistical significance of associations between categorical variables of the demographic factors (age, gender, educational status, computer literacy, previous exposure to research) and other items in the questionnaire (pharmacist’s attitude, knowledge and skills). Results were considered statistically significant at *p* < 0.05.

A score was developed to assess pharmacists’ self-reported knowledge of 12 EBM terms (e.g. absolute risk, relative risk, meta-analysis, etc.): 1 point was assigned to the option “Some understanding”, 2 points were assigned to the option “Understand and could explain to others” and 0 points were given for other options: “Do not understand but would like to” and “Do not understand and not willing to know”. The total possible score was 24 and the cut-off was 12, representing the median score; ≥12 indicated “adequate” knowledge and <12 indicated “inadequate” knowledge.

The interviews were transcribed verbatim. Field notes were taken during and after the interviews to ensure that all important information were documented. A qualitative approach comprising of thematic analysis was undertaken for data analysis. A coding frame was developed based on the themes identified during the interviews. Subsequently, the data relating to each theme/subtheme were repeatedly revisited until it was evident that no new themes emerged.

## Results

### A. Results from the EBM questionnaire

#### Socio-demographic data

A total of 176 pharmacists (of 445) working in the secondary and tertiary government hospitals in Kuwait agreed to take part in the study, giving a response rate of 40%. Most of the pharmacists were non-Kuwaitis (*n* = 97, 55.1%); were female (*n* = 96, 54.9%); were aged 31–40 years (*n* = 72, 41.2%); had a B.Sc. degree in pharmacy as qualification (*n* = 135, 78.5%); had 1–5 years of experience (*n* = 79, 45.1%); and had the employment rank of a pharmacist (*n* = 70, 40%). Table 1 shows the socio-demographic factors in more detail.

**Table 1:** Socio-demographic factors and their associations with computer literacy

			Computer literacy			
Socio-demographic Factors		All (N = 176)	Basic or less (N = 43)	Good (N = 93)	Excellent (N = 40)	Chi-Square *P* - value
	n	(Total %)	n (Row %)	n (Row %)	n (Row %)	
Age group (years)						0.032
22–30	69	(39.4)	13 (18.8)	35 (50.7)	21 (30.4)	For trend
31–40	69	(41.2)	20 (27.8)	36 (50.0)	16 (22.2)	
Above 40	34	(19.4)	9 (26.5)	22 (64.7)	3 (8.8)	
Gender						0.867
Male	79	(45.1)	18 (22.8)	42 (53.2)	19 (24.0)	Pearson
Female	96	(54.9)	25 (26.0)	50 (52.1)	21 (21.9)	
Nationality						0.817
Kuwaiti	79	(44.9)	21 (26.6)	40 (50.6)	18 (22.8)	Pearson
Non-Kuwaiti	97	(55.1)	22 (22.7)	53 (54.6)	22 (22.7)	
Educational level						0.295
BSc	135	(78.5)	28 (20.7)	79 (58.6)	28 (20.7)	For trend
Pharm D	15	(8.7)	7 (46.7)	6 (40.0)	2 (13.3)	
Postgraduate	22	(12.8)	4 (18.2)	8 (36.3)	10 (45.5)	
Years of experience						0.364
1–5	79	(45.1)	20 (25.3)	38 (48.1)	21 (26.6)	For trend
6–15	70	(40.0)	16 (22.9)	38 (54.2)	16 (22.9)	
Above 15	26	(14.9)	7 (26.9)	16 (61.5)	3 (11.6)	
Rank of employment						0.476
Beginner pharmacist	32	(18.3)	7 (21.9)	14 (43.8)	11 (34.3)	For trend
Pharmacist	70	(40.0)	19 (27.1)	37 (52.9)	14 (20.0)	
Pharmacy specialist	26	(14.9)	7 (26.9)	13 (50.0)	6 (23.1)	
Senior pharmacist	24	(13.7)	5 (20.8)	12 (50.0)	7 (29.2)	
Senior pharmacy specialist	9	(5.1)	1 (11.1)	8 (88.9)	0 (0.0)	
Head of pharmacy specialists	14	(8.0)	4 (28.6)	8 (57.1)	2 (14.3)	
Previous exposure to EBM						0.025
Read about it	82	(48.0)	25 (30.5)	43 (52.4)	14 (17.1)	Pearson
Attended a lecture	32	(18.7)	5 (15.6)	16 (50.0)	11 (34.4)	
Had formal EBM training	18	(10.5)	2 (11.1)	7 (38.9)	9 (50.0)	
Never heard of it	39	(22.8)	9 (23.1)	24 (61.5)	6 (15.4)	
Previous research exposure						0.105
Participated in one study	48	(28.1)	9 (18.8)	31 (64.6)	8 (16.6)	Pearson
Have participated in more than one study	20	(11.7)	6 (30.0)	6 (30.0)	8 (40.0)	
None	103	(60.2)	27 (26.2)	52 (50.5)	24 (23.3)	

The majority of the study respondents (75.5%) rated themselves as computer literate: 52.8% had good computer skills and 22.7% had excellent computer skills. Assessment of the association between computer literacy and the pharmacists’ demographics revealed that there was a significant indirect association with age (*p* = 0.032). The percent of pharmacists with excellent computer literacy within the youngest age group was higher (22 to 30 years; 30.4%) than that of the oldest age group (>40 years; 8.8%). There was also a significant association between computer literacy and previous exposure to EBM (*p* = 0.025), showing that 50% of those who had received formal EBM training (*n* = 18, 10.5%) had excellent computer literacy.

With regard to previous exposure to EBM, it was observed that 66% (*n* = 114) of the study sample had either read about EBM or attended a lecture. This was significantly associated with gender, nationality and previous research exposure (*p* = 0.043, 0.008, <0.001, respectively). Within this context, females were more likely to choose “at least read about it” (51.9%), while those who had never heard about EBM were mostly males (56.4%). In terms of nationality, most of the Kuwaiti pharmacists had attended a course or had heard about EBM, while 66.7% of the non-Kuwaitis had never heard of it. Also, the results showed that previous research exposure in terms of participating in one study (*p* < 0.001) was high among the groups who had attended a lecture on EBM or who had received formal EBM training (59.4% and 33.3%, respectively).

#### Pharmacists’ knowledge of EBM

This section aims at assessing the pharmacists’ perceived knowledge and understanding with regard to the basic terms used in EBM (see Table 2). Slightly more than half of the pharmacists (*n* = 85, 55.2%) had inadequate knowledge of EBM terms (score < 12). However, the respondents who did not understand these terms showed willingness to learn.

**Table 2:** Percentage of pharmacists’ knowledge about EBM terms

EBM Knowledge Term	Do not understand and not willing to learn	Do not understand but would like to learn	Some understanding	Understand and could explain to others
Relative risk	4 (2.3%)	29 (16.8%)	82 (47.4%)	58 (33.5%)
Absolute risk	5 (2.9%)	32 (18.5%)	62 (35.8%)	74 (42.8%)
Systematic review	5 (2.9%)	55 (32.0%)	68 (39.5%)	44 (25.6%)
Odds ratio	6 (3.5%)	80 (46.5%)	59 (34.3%)	27 (15.7%)
Meta-analysis	8 (4.7%)	76 (44.7%)	54 (31.8%)	32 (18.8%)
Clinical effectiveness	4 (2.3%)	25 (14.5%)	74 (42.8%)	70 (40.4%)
Sample size calculation	3 (1.7%)	43 (24.9%)	76 (43.9%)	51 (29.5%)
Confidence interval	3 (1.8%)	69 (42.1%)	61 (37.2%)	31 (18.9%)
*P*-value	7 (4.1%)	73 (42.9%)	54 (31.8%)	36 (21.2%)
Heterogeneity	9 (5.3%)	69 (40.6%)	56 (32.9%)	36 (21.2%)
Publication bias	7 (4.2%)	68 (40.5%)	48 (28.6%)	45 (26.7%)
Sensitivity	4 (2.3%)	48 (28.1%)	58 (33.9%)	61 (35.7%)

All of the socio-demographic variables were not significantly associated with EBM knowledge scores (*p* ≥ 0.05), except for the item “previous exposure to EBM” (*p* = 0.001).

#### Pharmacists’ self-reported skills for practising EBM

**Fig. 1:**
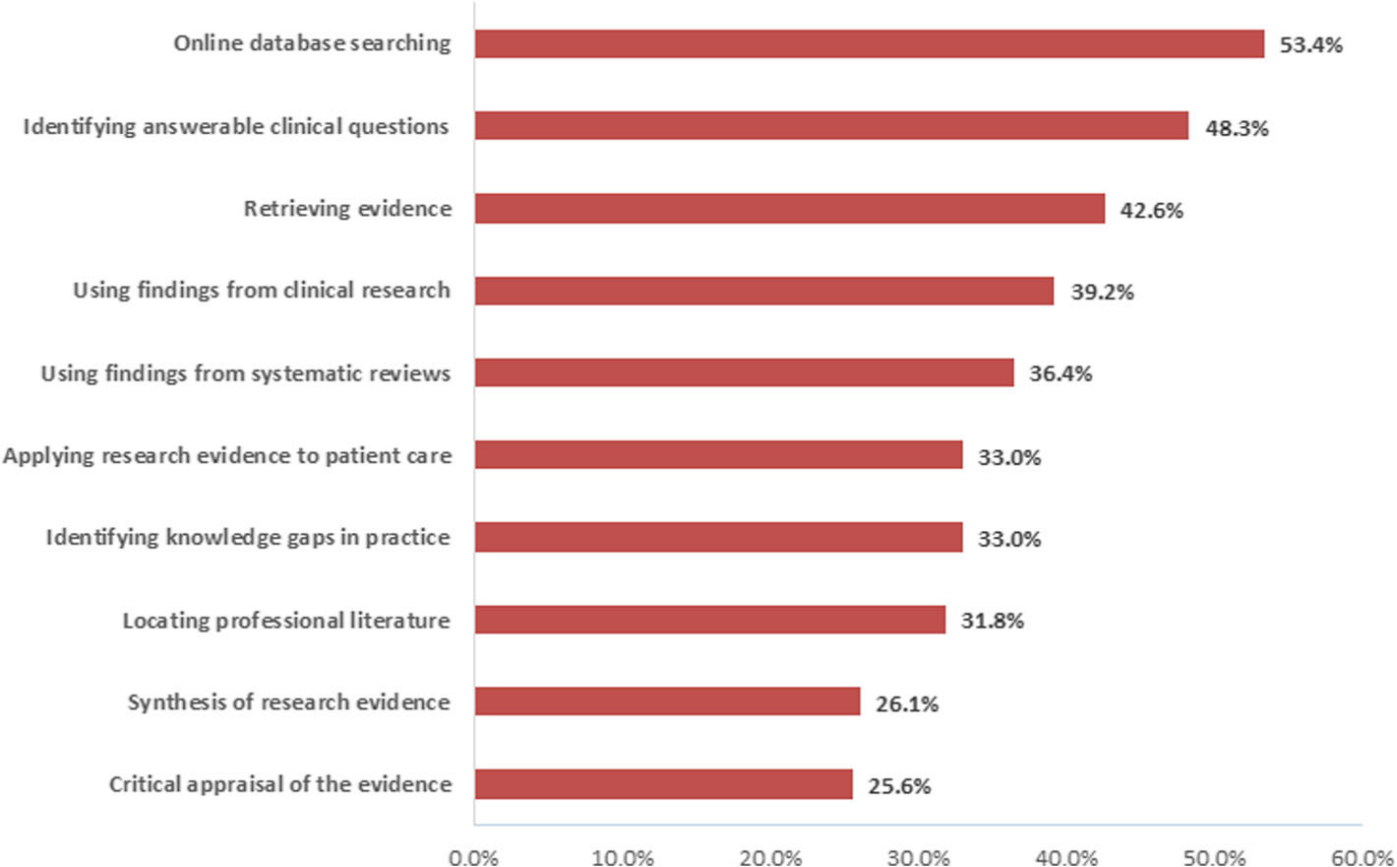
The pharmacists’ self-reported confidence skills required for practising EBM

Figure 1 shows the pharmacists’ self-reported confidence in the skills required for practising EBM. Over half of the study sample had good confidence in performing online database searches (*n* = 94, 53.4%) and identifying answerable clinical questions (*n* = 85, 48.3%), followed by retrieving evidence from the literature (*n* = 75, 42.6%). In contrast, their skills in performing more-complex tasks such as synthesising research evidence (*n* = 46, 26.1%) or critically appraising research evidence (*n* = 45, 25.6%) were limited. With regard to clinical practice, a third of the pharmacists reported that they had the capability to apply research evidence to patient care or to identify knowledge gaps in practice, whereas 67% of them did not have such skills.

**Fig. 2:**
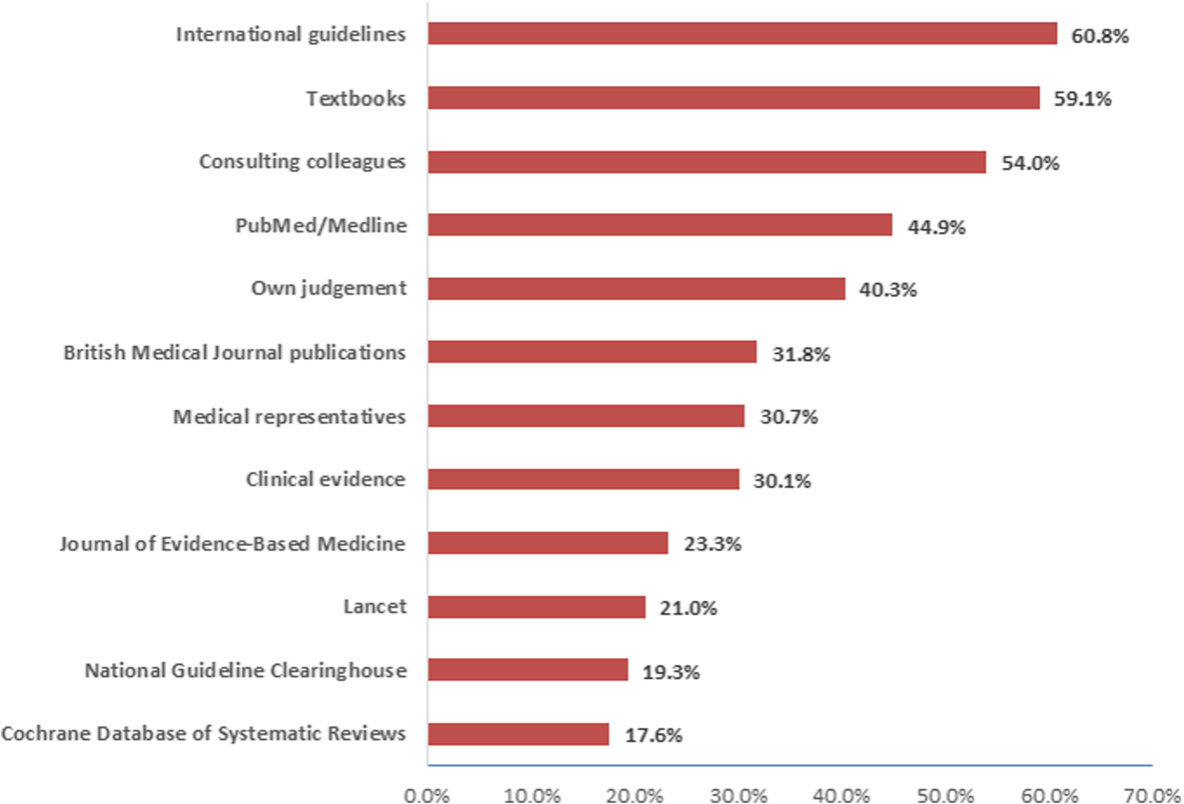
The Pharmacists’ use of varied resources

Regarding the various EBM resources, the most common resources used by the pharmacists to search for clinical evidence were international guidelines (*n* = 107, 60.8%), textbooks (*n* = 104, 59.1%) and colleagues (*n* = 95, 54%) (Fig. 2). More than a third of the respondents however, relied on PubMed/Medline (*n* = 79, 44.9%) or their own judgement (*n* = 71, 40.3%) to take a decision concerning patient care.

#### Attitude and opinions of pharmacists towards EBM use in clinical practice

**Fig. 3:**
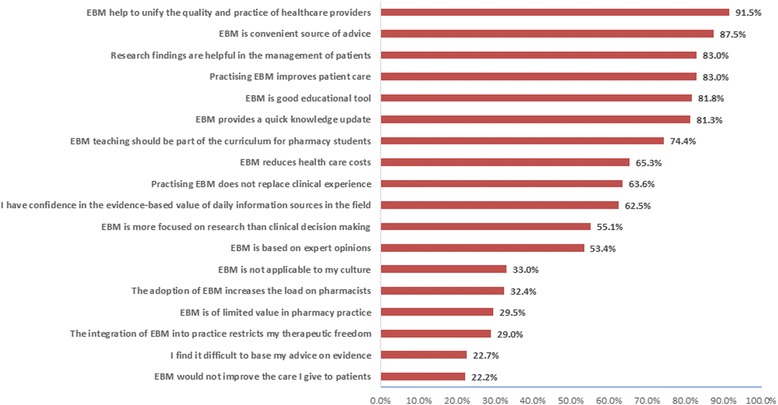
Attitudes and opinions of pharmacists towards EBM use in pharmacy practice

The majority of the pharmacists showed a positive attitude towards using EBM in their daily practice (Fig. 3). More than 80% of the pharmacists believed that EBM improves the quality of care provided (*n* = 161); helps in the management of patient cases (*n* = 146); improves patient care (n = 146); provides a quick knowledge update (*n* = 143); is a good educational tool (*n* = 144); and is a convenient source of advice (*n* = 154). On the other hand, almost a third of the pharmacists thought that EBM was not applicable to their culture (*n* = 58) and that its adoption in clinical practice would increase their daily workloads (*n* = 57). Around 29% of the pharmacists (*n* = 52) thought that EBM was of limited value in pharmacy practice and would restrict their therapeutic freedom (*n* = 51), while 22% of them believed that it is difficult to base a pharmacist’s advice on evidence (*n* = 40) and that EBM would not improve the delivery of care to patients (*n* = 39).

#### Barriers and facilitators in practising EBM among pharmacists

**Fig. 4:**
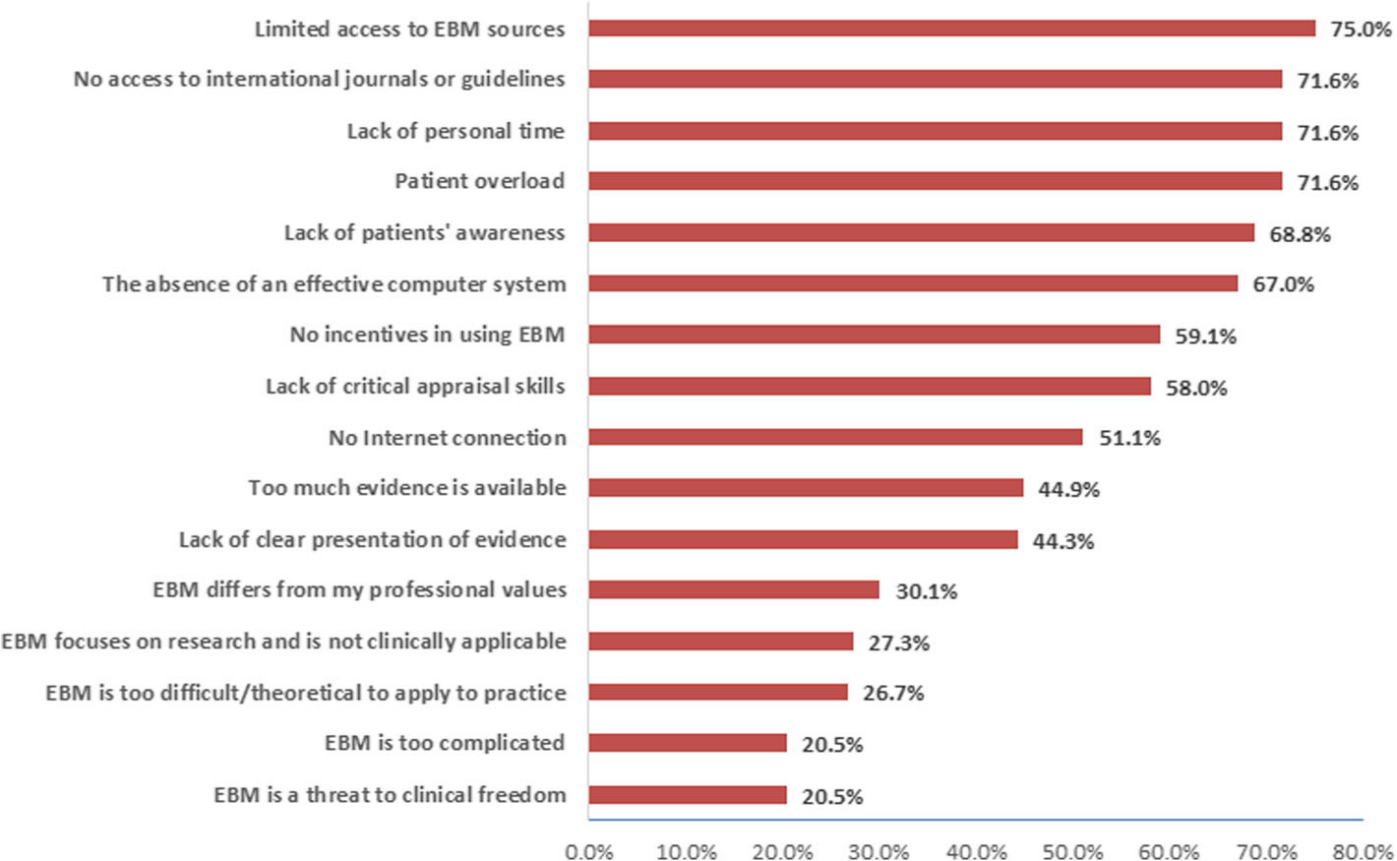
The Pharmacists’ barriers to incorporate EBM in pharmacy practice

The most common barriers to implementing EBM included limited access to EBM resources (*n* = 132); no access to international journals and guidelines (*n* = 126); a lack of personal time (n = 126); a lack of patient awareness (*n* = 121); and patient overload (n = 126) (Fig. 4). Also, the lack of incentives for pharmacists (*n* = 104) was regarded as a discouraging factor.

**Fig. 5:**
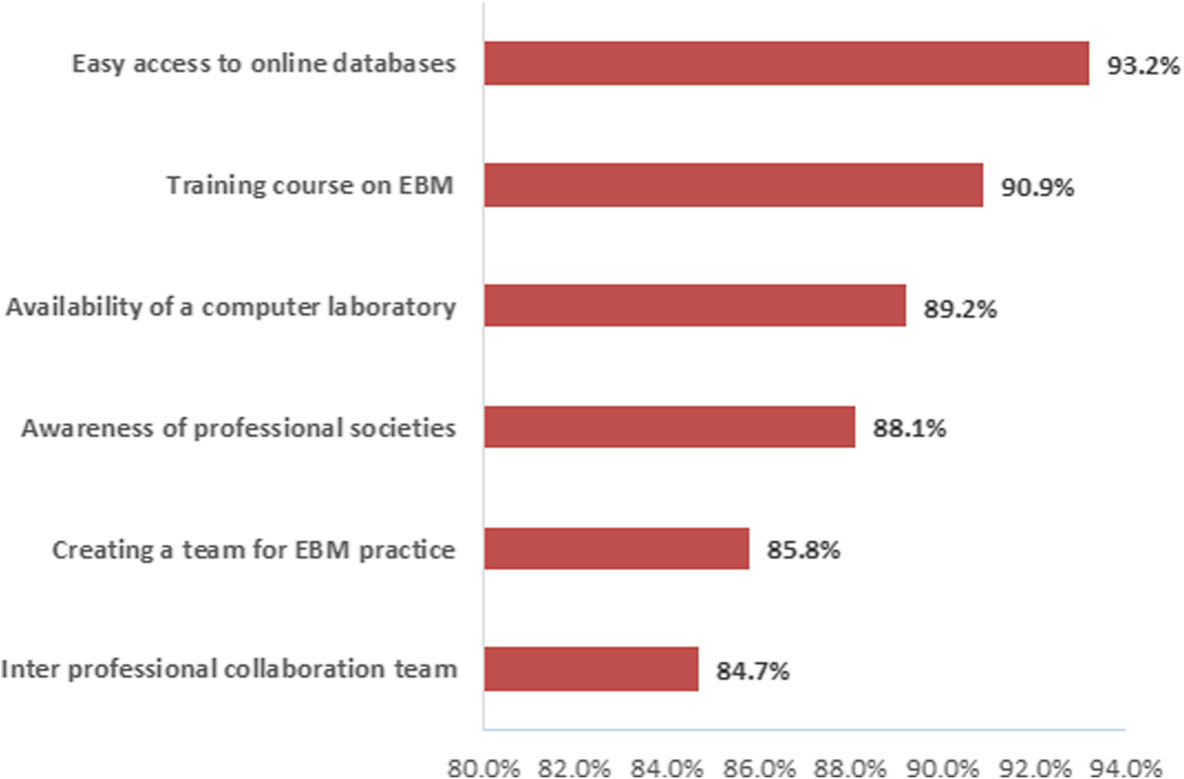
The most Facilitators for implementing EBM in pharmacy practice

Most of the pharmacists agreed that EBM in pharmacy practice could be facilitated by promoting awareness of EBM among professional societies (*n* = 155), increasing the availability of suitable resources (e.g. computer laboratories; *n* = 157), free access to online databases (*n* = 146), EBM training courses (*n* = 160) and collaborative teams (*n* = 149) (Fig. 5).

### B. Results from the semi-structured interviews

The participants’ responses to the semi-structured questions were grouped into three themes: the overall concept of EBM, the adoption of EBM in pharmacy practice, and factors that facilitate or hinder the implementation of EBM in pharmacy practice in Kuwait. Under each theme, several sub-themes were introduced.

The interviewees were in the age range between 35 and 45 years, except one, who was 55 years old. All of the interviewees held a B.Sc. degree in pharmacy with 10–15 years of experience in pharmacy practice. The mean interview duration was 20 min.

#### Overall concept of EBM

None of the interviewees (*n* = 6) were aware about EBM and its practice; however, two of them had heard about the concept of EBM but lacked the knowledge and skills on how to practise it. One of the interviewees’ responses was “Well, I haven't heard about it as a concept (EBM)…could you explain it?…Does it need a specific knowledge for practising it?”.

#### Adoption of EBM in pharmacy practice in Kuwait

During the interviews, the chief pharmacists were asked about their perceptions regarding incorporating EBM into the clinical practice of pharmacy in Kuwait and the readiness of pharmacists in Kuwait to undertake EBM practices.

### Willingness to introduce EBM into pharmacy practice

The interviews revealed that all of the chief pharmacists (*n* = 6) were willing to incorporate EBM into pharmacy practice. One stated that *“*Evidence-based practice should be an integral part of pharmacy practice”. All of the interviewees agreed that pharmacists working in different government hospitals are generally eager to improve their professional image, particularly newly graduated pharmacists, who are passionate about pharmacy. One of the interviewees reported: *“*…we believe in our staff….there are pharmacists specifically fresh graduates who are working and attempting to improve pharmacy image as a profession that has an important role in patient care.” The interviewees’ attitude towards introducing changes and improvements in the pharmacy profession were found to be positive during the interviews.

All of the chief pharmacists (*n* = 6) supported the incorporation of EBM into pharmacy practice. They emphasized that pharmacists should play a vital role in providing pharmaceutical care, and this care should be the best and most up to date: “EBM is an important practice not for pharmacists alone but for all healthcare providers, for whom it is important to stay current with the latest treatments and techniques.”

### Readiness for introducing EBM into pharmacy practice

The readiness of the pharmacy administration to adopt EBM in pharmacy practice was addressed during the interviews with the chief pharmacists. Almost all of the interviewees (*n* = 5) agreed that readiness mainly requires support from the decision-makers in the MOH. Consequently, all the requirements for EBM practice, including electronic resources and training courses for the pharmacists, would be offered. One of the chief pharmacists stated that “our readiness to practise EBM can be improved in many ways, but the main issue is the higher management’s support (financial and technological), as pharmacists’ willingness and attitudes can be enhanced afterward”.

#### Current factors that facilitate/hinder the practice of EBM in Kuwait

The majority of the interviewees (n = 5) stated that support from the MOH decision-makers and higher management was the most important factor that could overcome the challenges towards implementing EBM in clinical practice. One of the interviewees stated: “an official decision from the higher management is needed to start working evidence-based…this would be a good start-up for all the staff in which computers and internet access will be provided officially.”

The chief pharmacists believed that pharmacy staff members usually work as a team, in which the work values are directed towards patients care. In this respect, they did not think that introducing EBM into practice would face individual resistance from the pharmacists. One of the interviewees stated: “Our culture is not against EBM practice; on the contrary, we accept the changes in our practice for the patients’ sake.” All the interviewees agreed on the importance of raising the awareness of EBM and providing EBM training courses and management support to improve pharmacists’ attitude towards practising EBM. Some also stated that patients also need to be aware of this evolution of the pharmacists’ role in order to maintain their confidence in the pharmacists. One interviewee reported “…even patients they need to be aware about this practice so they will not refuse the evidence being presented to them and to be confident towards the pharmacists' practice.”

All of the chief pharmacists agreed that technological resources would be needed for practising EBM. However, it was evident from the interviews that one of the current barriers that will delay the adoption of EBM in pharmacy practice is the lack of technological resources in the hospitals. These resources include computer laboratories/libraries, Internet connections, and free access to different medical and pharmaceutical journals and online databases: “The most significant barrier to EBM practice in our hospitals is the lack of online medical database subscriptions”.

Beside the technological resources, managerial and administrative issues were mentioned by the chief pharmacists as crucial, such as setting up a policy to support the practice of EBM, as well as giving pharmacists the authority to arrange committees and develop clinical practice guidelines based on new clinical evidence. As reported by one interviewee: “Incorporating EBM into pharmacy practice requires developing a new policy to organise the work among the pharmacy staff”.

Moreover, one of the motivating factors for practising EBM mentioned by most of the interviewees (*n* = 4) was offering financial incentives to EBM practitioners as encouragement.

## Discussion

This study found that there was a significant association between the pharmacists’ computer literacy and socio-demographic factors, such as age and previous exposure to EBM, showing that: [1] the younger pharmacists were more competent in computer skills; and [2] the pharmacists with more exposure to EBM were more competent in computer skills. These findings could be explained by the fact that academic institutions are using computers and informatics in their current educational curricula, which likely has a positive impact on their graduates [27]. Additionally, the wide use of smart technology in society could have an impact on computer skills among the younger generation in general. The study also found that exposure to EBM training is important, as it leads to having hands-on computer skills in searching medical databases and locating research evidence.

The findings from the current study revealed that the pharmacists who had published at least one article were more knowledgeable about EBM practice. This could be explained by the dependence of EBM on research and literature searching skills. Furthermore, most of the Kuwaiti pharmacists had attended a course or had heard about EBM, whereas a majority of non-Kuwaitis (66.7%) had never heard of it. This could be a result of individual efforts to improve the knowledge in general. Also, the work experience of the pharmacists after graduation could have a strong influence in this regard. Our results are similar to another study of primary care physicians in Kuwait which reported that, non-Kuwaiti physicians had a lower awareness of EBM, as compared to their Kuwaiti peers [23].

### Knowledge and skills required to practise EBM

The present study also found that the pharmacists’ EBM skills varied: roughly 50% of the respondents reported that they are familiar with searching the Internet for medical resources, asking answerable clinical questions and retrieving research evidence. However, the pharmacists reported a lack of skills to synthesise evidence from the literature and to critically appraise research articles. Furthermore, the results showed that 67% of the pharmacists were unable to apply research evidence to patient care or to identify knowledge gaps in practice. These findings are consistent with those of previous studies, which highlighted that a lack of knowledge and skills to apply EBM into practice were considered a barrier [16, 17, 23, 28].

There was some understanding among the pharmacists in the current study in regard to EBM research terms (e.g. relative risk, absolute risk, clinical effectiveness and sensitivity). Not surprisingly, the majority of the pharmacists lacked knowledge of specific statistical terminology (e.g. odds ratio, *p*-value, confidence interval, heterogeneity and bias), as these terms are more common among practitioners who are dedicated to research. However, the respondents expressed a willingness to learn. Previous studies among physicians have also reported a low level of knowledge of EBM-related terms [7, 12, 29]. A good EBM practitioner should be able to read and evaluate research critically, both of which depend on understanding statistical terms clearly so that the best current clinical evidence can be selected.

Regarding previous exposure of the pharmacists to EBM knowledge, this study found that attending a training course or a one-day session had an influence on the pharmacists’ knowledge of EBM terms. These findings are consistent with the results of a previous study among Brazilian community pharmacists [25] but are not consistent with a study in Iran, which reported that physicians did not show improved EBM knowledge after undertaking an EBM training course [11]. Other studies have confirmed the importance of EBM training courses on practitioners’ knowledge, which leads to better understanding, and therefore, better pharmaceutical care for patients [7, 17, 28]. However, it appears it will be difficult to sustain a change in practice through only offering training courses if other key barriers remain, such as lack of support, time restrictions, and internet access.

The current study found that most of the pharmacists relied on colleague consultation (54%), textbooks (59.1%) and international guidelines (60.8%) when they sought information, followed by using online medical resources, such as PubMed/Medline. Using the Cochrane Database was the least common source of information (17.6%). However, colleagues’ advice and textbooks may not be evidence-based or up-to-date as information sources. It appears that electronic resources were not officially available to the pharmacists in the hospitals, and more than half of participants reported that they are deficient in their awareness of these electronic databases and how they could utilise them. The semi-structured interviews with the chief pharmacists supported these findings and confirmed that there is a lack of resources available to the pharmacists to enable them to conduct literature searches (e.g. unavailability of computer laboratories with Internet connections or subscriptions to online databases). Similar findings were found with family physicians in Kuwait [23], who frequently relied on their own judgements (68.6%) and medical books (52.8%) to make clinical decisions. These findings are supported by the results reported by other regional studies conducted in Saudi Arabia and Qatar [29]. Furthermore, previous studies regarding EBM in pharmacy practice have shown that in Jordan [7], pharmacists relied mostly on their own judgements in giving pharmaceutical information (80%), in addition to medical representatives’ information (72%) and medical textbooks (54.5%), rather than EBM processes. Another study in Brazil [25], reported that community pharmacists relied on searching Google (62.2%), using textbooks (49.5%) and consulting colleagues (37%) to obtain information. It is clear that a majority of healthcare practitioners rely on traditional resources when seeking information, which is different from the principles of EBM.

### **Attitude and opinions of pharmacists towards EBM use in clinical practice**

The majority of the pharmacists showed a positive attitude and willingness to use EBM in their clinical practice. This was also consistent with the perspectives of the chief pharmacists, who reported thinking that hospital pharmacists, particularly the new graduates, have the greatest willingness and energy to improve pharmacy practice by adopting EBM. The pharmacists in the current study believed that EBM helps in patient case management, patient care improvement and knowledge updating and is a good educational tool and a convenient source of advice. These findings are consistent with those of a study conducted in Jordan [7].

On the contrary, less than a third of the respondents in the current study thought that introducing EBM into pharmacy practice would limit the freedom of medication choice, thereby negatively affecting clinical practice in general. This reflects the misunderstanding of these pharmacists regarding a core principle of EBM, which is to inform the practitioner about new medicines that could differ from those in conventional guidelines. This in turn could make clinical decisions easier and more accurate through being evidence based. It is also important for the pharmacist to be competent in using EBM to avoid pharmacotherapy malpractice and undesirable clinical outcomes.

### **Barriers and facilitators in practising EBM among pharmacists**

Eliminating barriers requires identifying them first [10]. The findings of this study identified the barriers that would limit today’s hospital pharmacists in Kuwait from practising EBM. The most common hindrances were a lack of access to online medical resources (including international journals), a lack of computer facilities, a lack of time and work overload. Similarly, in the interviews, the unavailability of supportive resources for practising EBM was mentioned as the main barrier, besides the lack of higher management support for a formal decision to incorporate EBM into practice, in which all barriers would be eliminated accordingly. Similar barriers were found in many other professions, including medicine [29], nursing [5] and physical therapy [30].

‘Culture’ was not considered a major barrier to the practice of EBM among the pharmacists in the government hospitals in Kuwait. This was indicated by the pharmacists responses and further reported by the chief pharmacists during the interviews. Similar findings were found in a previous study conducted in Dubai [13]. It appears that culture has little influence on the pharmacists attitude towards EBM in clinical environments, with the quality of healthcare being the crucial factor.

Several solutions were suggested by the pharmacists to facilitate the practice of EBM in the hospitals. Most importantly, raising awareness of EBM among professional societies would improve teamwork within the pharmacies and with other healthcare professionals, which would improve the quality of patient care overall. One of the main EBM facilitators was the availability of resources, which included computer laboratories, Internet connections and free access to online databases. Furthermore, the respondents considered EBM training courses as important, viewing them as effective in improving the EBM knowledge and awareness of the practitioners. These encouraging or facilitating factors were addressed in previous studies [2, 13, 31, 32]. Moreover, the results showed that organising teamwork for EBM is considered a crucial point in introducing EBM into pharmacy practice. The interview results complemented the results of the questionnaire about facilitating EBM practice in pharmacy, focusing on three important organisational issues: [1] developing a policy for practising EBM that organises, standardises and facilitates clinical teamwork; [2] offering incentives to EBM practitioners as encouragement; and lastly and most importantly, [3] obtaining a decision from the MOH to invest in the resources needed to incorporate EBM into clinical practice. Thereafter, all relevant resources would be offered accordingly.

### Limitations

- Despite assuring the confidentiality and anonymity of the data, the interviewees preferred not to digitally audio-record the interviews. They also refused the idea of storing the recordings with the researcher even for research purposes.
- Due to the busy work schedule of the pharmacists in the hospitals, some of them refused to take part in the study, which contributed to the low response rate. The length of the questionnaire (85 items) may have been another reason for the low response rate in the study.
- The questionnaire was only prepared in English since the majority of pharmacists had completed their pharmacy training in English. However, it seemed that some of the pharmacists had graduated from programs in which the academic education is delivered in Arabic, and were not able to complete the English Questionnaire.
- Lastly, the study did not enroll pharmacists from private hospitals, since it did not aim to compare the knowledge, attitude and practices of government versus private hospital pharmacists. Therefore, these study results cannot be generalized to pharmacists at private hospitals in Kuwait.

## Conclusion

The hospital pharmacists in Kuwait reported a good attitude and willingness to practice EBM. However, they need to acquire adequate knowledge and skills to apply it in “real life” practise. From the overall findings, results showed that barriers do exist and hinder the practise of EBM. Therefore, in order to provide evidence-based pharmaceutical care, support is needed from policy makers and higher management in the Kuwait MOH. In this regard, clinical implications were recommended to demonstrate how to overcome the barriers, wherein hospital pharmacists could be ready to practice EBM.

### Clinical implications

The results from the current study could have a great impact on improving clinical practice through the following recommendations:

- The Kuwait Pharmaceutical Association (KPA) should organise regular awareness seminars, distributing brochures and exhibiting roll-up banners in healthcare organisations to emphasize the importance of EBM in clinical practice.
- Providing management support at all levels, including providing computer facilities, printers and free access to online medical resources at the point of care. Also, giving pharmacists the authority to hold committees specifically to assess the clinical evidence found and to confirm the new changes and clinical guidelines.
- Building a pharmaceutical team that can cope with the constant changes in medical care by keeping abreast with up-to-date information with the goal of providing the best clinical practice and quality care to patients. This could be done by assigning three pharmacists to conduct ‘rapid reviews’ of the medical literature on a monthly basis.
- Developing policy and guidelines for EBM in pharmacy practice and to organise the pharmaceutical team and standardise the work within the department.
- Encouraging inter-professional collaboration, particularly among physicians and pharmacists, which would allow healthcare professionals to share clinical practice in determining the best treatment plans for medical cases.
- Arranging training courses/workshops by the KPA for pharmacists, physicians and other allied health professionals, focusing on the depth of knowledge of EBM and hands-on practice.
- Offering incentives at the initial stage of the practice to encourage pharmacists to render more effort to adopt and maintain good EBM practice. Adopting an EBM approach in pharmacy practice is considered a forward-thinking and crucial step in improving pharmaceutical care, and in reducing medical errors and improving the safety of pharmaceutical practice.
- Teaching the future generations of pharmacists the principles of EBM that need to be applied in pharmacy practice. The Faculty of Pharmacy, Kuwait University should incorporate an EBM module into the curriculum of the Bachelor Degree. To ensure best academic outcomes, course evaluation by students should be considered.

## Abbreviations

(KPA): The Kuwait Pharmaceutical Association
AACP: The American Association of Colleges of Pharmacy
EBM: Evidence-Based Medicine
MoH: Ministry of Health
